# Combination of Whole-Brain Radiotherapy with Epidermal Growth Factor Receptor Tyrosine Kinase Inhibitors Improves Overall Survival in EGFR-Mutated Non-Small Cell Lung Cancer Patients with Brain Metastases

**DOI:** 10.3390/cancers11081092

**Published:** 2019-07-31

**Authors:** Chien-Hung Chen, Hsin-Hua Lee, Hung-Yi Chuang, Jen-Yu Hung, Ming-Yii Huang, Inn-Wen Chong

**Affiliations:** 1 Department of Radiation Oncology, Kaohsiung Municipal Ta-Tung Hospital, Kaohsiung 80145, Taiwan; 2 Graduate Institute of Medicine, College of Medicine, Kaohsiung Medical University, Kaohsiung 80708, Taiwan; 3 Ph.D. Program in Environmental and Occupational Medicine, Kaohsiung Medical University and National Health Research Institutes, Kaohsiung 80708, Taiwan; 4 Department of Radiation Oncology, Kaohsiung Medical University Hospital, Kaohsiung Medical University, Kaohsiung 80708, Taiwan; 5 Department of Occupational and Environmental Medicine, Kaohsiung Medical University Hospital, Kaohsiung Medical University, Kaohsiung 80708, Taiwan; 6 Division of Pulmonary and Critical Care Medicine, Department of Internal Medicine, Kaohsiung Medical University Hospital, Kaohsiung Medical University, Kaohsiung 80708, Taiwan; 7 School of Medicine, College of Medicine, Kaohsiung Medical University, Kaohsiung 80708, Taiwan; 8 Department of Radiation Oncology, Faculty of Medicine, College of Medicine, Kaohsiung Medical University, Kaohsiung 80708, Taiwan; 9 Center for Drug Development and Value Creation Research Center, Kaohsiung Medical University, Kaohsiung 80708, Taiwan; 10 Center for Cancer Research, Kaohsiung Medical University, Kaohsiung 80708, Taiwan; 11 Department of Respiratory Therapy, Faculty of Medicine, College of Medicine, Kaohsiung Medical University, Kaohsiung 80708, Taiwan

**Keywords:** brain metastases, epidermal growth factor receptor, non-small cell lung cancer, whole-brain radiotherapy, tyrosine kinase inhibitors

## Abstract

Brain metastases (BM) cause morbidity and mortality in patients with non-small cell lung cancer (NSCLC). The use of upfront epidermal growth factor receptor (EGFR) tyrosine kinase inhibitors (TKIs) and withholding of whole-brain radiation therapy (WBRT) is controversial. We aim to investigate the impact of WBRT on overall survival (OS). After screening 1384 patients, a total of 141 EGFR-mutated patients with NSCLC and BM were enrolled. All patients received EGFR-TKIs between 2011 and 2015. Ninety-four patients (66.7%) were treated with WBRT (TKI + WBRT group). With a median follow-up of 20.3 months (95% confidence interval (CI), 16.9–23.7), the median OS after the diagnosis of BM was 14.3 months (95% CI, 9.5 to 18.3) in the TKI + WBRT group and 2.3 months (95% CI, 2 to 2.6) in the TKI alone group. On multivariate analysis, WBRT (*p* < 0.001), female, surgery to primary lung tumor, and surgery to BM were associated with improved OS. The 1-year OS rate was longer in the TKI+WBRT group than that in the TKI alone group (81.9% vs. 59.6%, *p* = 0.002). In conclusion, this is the first study to demonstrate the negative survival impact from the omission of WBRT in patients with EGFR-mutant NSCLC.

## 1. Introduction

Globally, lung cancer is a leading cause of cancer incidence and mortality [1], and is also the most common primary site of brain metastases (BM) [2]. The mainstay of treatment for BM has been surgery, whole-brain radiotherapy (WBRT) or stereotactic radiosurgery performed alone or in combination. WBRT targets any micro-metastases not detected on imaging, prevents intracranial recurrence, and reduces the risk of deaths due to neurological cause [3]. Because BM causes neurological decline, WBRT improves neurological function with minimal complications [4,5]. In 1954, it was reported to lessen headache, aphasia, hemiplegia, paralysis, blurred vision and incontinence [4]. On the other hand, radiotherapy (RT)-induced dementia in patients cured of BM was 1.9 to 5.1% [6]. 

Post-operative WBRT is usually recommended to prevent local recurrence and death from neurologic cause [7]. With the advance of modern treatment and improved cancer survival, the cognitive effect of WBRT is now a concern [8]. Even though the European Organization for Research and Treatment of Cancer (EORTC) 22952-26001 study revealed that post-operative adjuvant WBRT reduces intracranial relapses and neurologic deaths [9], another EORTC Phase-III trial reported an induced inferior quality of life [10]. A secondary analysis of EORTC 22952-26001 found no significant survival benefit to WBRT among patients with non-small cell lung cancer (NSCLC) and favorable Graded Prognostic Assessment (GPA) scores [11].

NSCLC accounts for approximately 85% of lung cancers [12]. It is characterized by a high incidence of BM. Advances in the understanding of genetic aberrations associated with NSCLC have led to the development of epidermal growth factor receptor (EGFR)-targeted therapies [12]. Patients with EGFR-mutated lung cancer tend to have longer survival rates, but a higher incidence of brain metastases [13]. WBRT alone is now the treatment of choice for patients who are poor candidates for surgery, yet a recent randomized controlled trial suggested no benefit of WBRT to optimal supportive care with dexamethasone in patients with poor performance status and multiple BM from NSCLC [14]. The fast-paced development of novel agents is allowing improved outcomes for patients with advanced NSCLC. Whether deferring WBRT compromises neurological and survival outcome is still under debate [15].

The optimal management of EGFR-mutated NSCLC with BM continues to develop, with new approaches to diagnosis and a continual expansion of available treatment options for patients with EGFR mutation. Current studies have reported that EGFR-mutated NSCLC exhibits better efficacy of RT than does EGFR wild-type [16,17,18]. Cell studies have proven that clonogenic survival of EGFR-mutated NSCLCs in response to RT was reduced 500- to 1000-fold compared with wild-type [19]. WBRT affects the blood-tumor barrier [20,21,22,23], and blood-brain barrier (BBB) disruption after WBRT treatment is documented to lead to an increase in drug permeability. Furthermore, TKI can increase the radiosensitivity of EGFR-mutated cells [23]. TKIs have different capacity to cross the BBB. In one study, the cerebrospinal fluid (CSF)-to-plasma ratio of gefitinib in patients with BM was higher than that in patients without BM (1.34% vs. 0.36%, *p* < 0.001) [24], while another study found that the BBB permeation rate of erlotinib was 4.4 ± 3.2% [25]. Hoffknecht et al. reported data from one patient with an impressive response showed an afatinib concentration in the CSF of nearly 1 nMol [26]. In the BLOOM study, the BBB permeation rate of osimertinib was found to be 16% [27].

The efficacy of TKIs with and without WBRT has not been determined in patients with BM resulting from EGFR-mutated NSCLC; thus, we aimed at identifying optimal therapeutic strategies for patients with BM in the setting of dominant oncogenic-driven disease. We hypothesized that WBRT in addition to TKI alone may offer survival benefits under the radiobiological rationale of the impact of WBRT on BBB permeability. To address this issue, we assessed the effectiveness of TKI given alone or in combination with WBRT to patients with EGFR-mutated NSCLC and newly diagnosed BM. 

## 2. Patients and Methods

### 2.1. Ethics Approval Statement

The present study (KMUHIRB-E(II)-20180185) was approved by the ethical and research committee of the Kaohsiung Medical University Hospital. This study was conducted in compliance with institutional review board regulations in accordance with the Helsinki Declaration of 1975 as revised in 1983. All patients provided written informed consent for treatment; patient information was anonymized and de-identified before analysis; consequently, all data were analyzed anonymously.

### 2.2. Patients

Of 1384 NSCLC patients in the database of the tertiary hospital, we identified and analyzed 141 consecutive patients with pathologically proven lung adenocarcinoma who had received TKIs between 3 January 2011 and 29 December 2015. Their BM were diagnosed by either cytology or brain neuroimaging studies. The inclusion criteria for this study were pathologically proven positive EGFR mutations, the diagnosis of BM, and the use of TKI. The exclusion criteria were a history of prior brain RT, or a history of malignancies other than lung cancer, or EGFR-TKI resistance mutation, or incapability to receive EGFR-TKI. Patient follow.ups were conducted by clinic visits or telephone calls until June 2018.

The following variables were collected: age, gender, initial clinical tumor and nodal classification, time from initial diagnosis to BM, extracranial metastasis, histological grading, EGFR mutation, operation to primary lung tumor, number of lines of chemotherapy, name of EGFR-TKI, number of lines of TKI, mean duration of TKI use, Eastern Cooperative Oncology Group (ECOG) performance status at the time of BM, number of BM, smoking history, whether the patient was symptomatic from BM, size of the largest BM, number of BM and disease-specific Graded Prognostic Assessment (dsGPA). The dsGPA was calculated for each patient to determine whether the cohorts shared similar prognostic features [28]. 

### 2.3. Target Therapy

All patients underwent pretreatment workups comprising a physical examination, a history review, chest radiography, bronchoscopy with a tumor biopsy, chest computed tomography (CT), brain magnetic resonance imaging (MRI) or CT, and routine laboratory studies. The tumor stage was classified according to the seventh edition of the Cancer Staging Manual and Handbook of the American Joint Committee on Cancer (AJCC) [29]. All patients started taking EGFR-TKI once the diagnosis of stages IIIB –IV lung cancer with EGFR mutation was established. TKIs included gefitinib, erlotinib, afatinib and osimertinib. The first generation was used and then shifting to the second or third generation might be chosen at the discretion of the thoracic oncologist.

### 2.4. WBRT

Ninety-four patients had WBRT once the diagnosis of BM was confirmed. For WBRT, each patient was simulated in the supine position in a customized thermoplastic immobilization mask. Three-dimensional conventional radiotherapy (3D-CRT) was delivered using a 2100 C/D linear accelerator (Varian Medical Systems, Palo Alto, CA, USA) for 57 patients. The remaining 37 patients were treated by intensity-modulated radiotherapy (IMRT) either with a Hi-Art helical tomotherapy unit, version 2.2.4.1 (TomoTherapy, Inc., Madison, WI, USA), or Eclipse, version 8.6 (Varian Medical Systems Inc., Palo Alto, CA, USA). For the 37 patients who had a boost dose to their BM, the tumor and boost beams were combined in one integrated treatment plan. Fractionation schemes were as follows: 30 Gy in 10 fractions with or without a simultaneous boost to the brain of 45 Gy, or 37.5 Gy in 15 fractions with or without a simultaneous boost to the brain of 45 Gy. The decision whether to give a RT dose boost to the BM sites was at the discretion of each radiation oncologist. The mean radiation dose was 3781 ± 749 cGy to BM.

### 2.5. Statistical Analysis

The primary end points were overall survival (OS) and the OS after a diagnosis of BM (OSm). OS was defined as the time from the date of lung cancer diagnosis to the date of death from any cause or until the date of the last follow-up. OSm was defined as the time from the date of BM diagnosis to the date of death from any cause or until the date of the last follow-up. OS and OSm rates were assessed by Kaplan–Meier methods and the log-rank test was used to compare time-to-event distributions. The data set was stratified and outcomes were compared by *t*-test or chi-squared test. Univariate analyses and a multivariate Cox proportional hazards regression were used to inspect all collected variables. Estimated risks of death were calculated using hazard ratios (HR) with 95% confidence intervals (CIs). The level of statistical significance was set at *p* < 0.05; all reported *p* values were two-tailed. The analyses were performed using the SPSS software package, version 19.0 for Windows (SPSS, Chicago, IL, USA).

## 3. Results

### 3.1. Patient Characteristics

One hundred forty-one patients out of 1384 patients were retrospectively enrolled after the aforementioned inclusion and exclusion criteria were applied. Gender difference existed in terms of smoking status (never vs. ever); 98.9% of the female patients and 37.7% of the male patients had never smoked (*p* < 0.001). The median duration of TKI use was 13.2 months (95% confidence interval (CI), 10.1 to 16.2) in TKI + WBRT group and 10 months (95% CI, 7.3 to 12.8) in the TKI alone group. The mean durations of TKI use were 18.1±15.1 months and 15.4 ± 16.4 months for patients with and without WBRT, respectively (*p* = 0.327). In this cohort of 141 patients, 52 patients had more than one line of TKIs due to intolerance or disease progression. Table 1 summarizes the clinical characteristics of the 141 patients, divided by whether they had WBRT (TKI + WBRT group vs. TKI alone group). 

All of them had EGFR-TKI. The mean and median age of this retrospective cohort was 64.5 years and 62 years respectively. Ninety-four patients (66.7%) received WBRT, and 47 patients (33.3%) did not. Patients who received WBRT were more likely to have surgery to their BM (38.3% in the TKI + WBRT group and 14.9% in the TKI alone group; *p* = 0.004); neurological symptoms (76.6% in the TKI + WBRT group and 53.2% in the TKI alone group; *p* = 0.005); larger BM (70.2% over 1 cm in the TKI + WBRT group and 53.2% in the TKI alone group; *p* = 0.046); and more BM (*p* = 0.043). No significant differences were observed in terms of age, gender, stage, initial clinical tumor and nodal classification, extracranial metastases, histological grading, EGFR mutation, primary lung surgery, number of lines of chemotherapy, name of EGFR-TKI, number of lines of TKI, mean duration of TKI use, smoking history, ECOG performance status at the time of BM, and dsGPA (all *p* > 0.05; Table 1).

### 3.2. OS and OSm

The median OS was 20.3 months (95% CI, 16.9 to 23.7) for the entire cohort. Seventeen and two patients were still alive in the TKI+WBRT group (18.1%) and TKI alone group (4.3%), respectively. The mean OS was longer for patients with WBRT (27.2 ± 16.7 vs. 21.6 ± 20.4 months, *p* = 0.033) 

The median OSm was 10.5 months (95% CI, 7.2 to 13.9) for the entire cohort. The combination group survived much longer after the diagnosis of BM. The median OSm was 14.3 months (95% CI, 9.5 to 18.3) in the TKI + WBRT group and 2.3 months (95% CI, 2 to 2.6) in the TKI alone group. The mean survival after BM was 18 ± 15.2 months and 7.1 ± 10.8 months for patients with and without WBRT, respectively (*p* < 0.001). 

**Figure 1:**
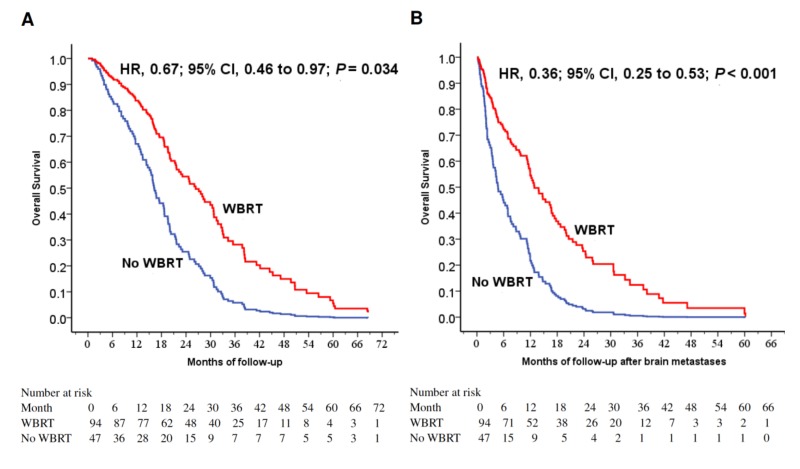

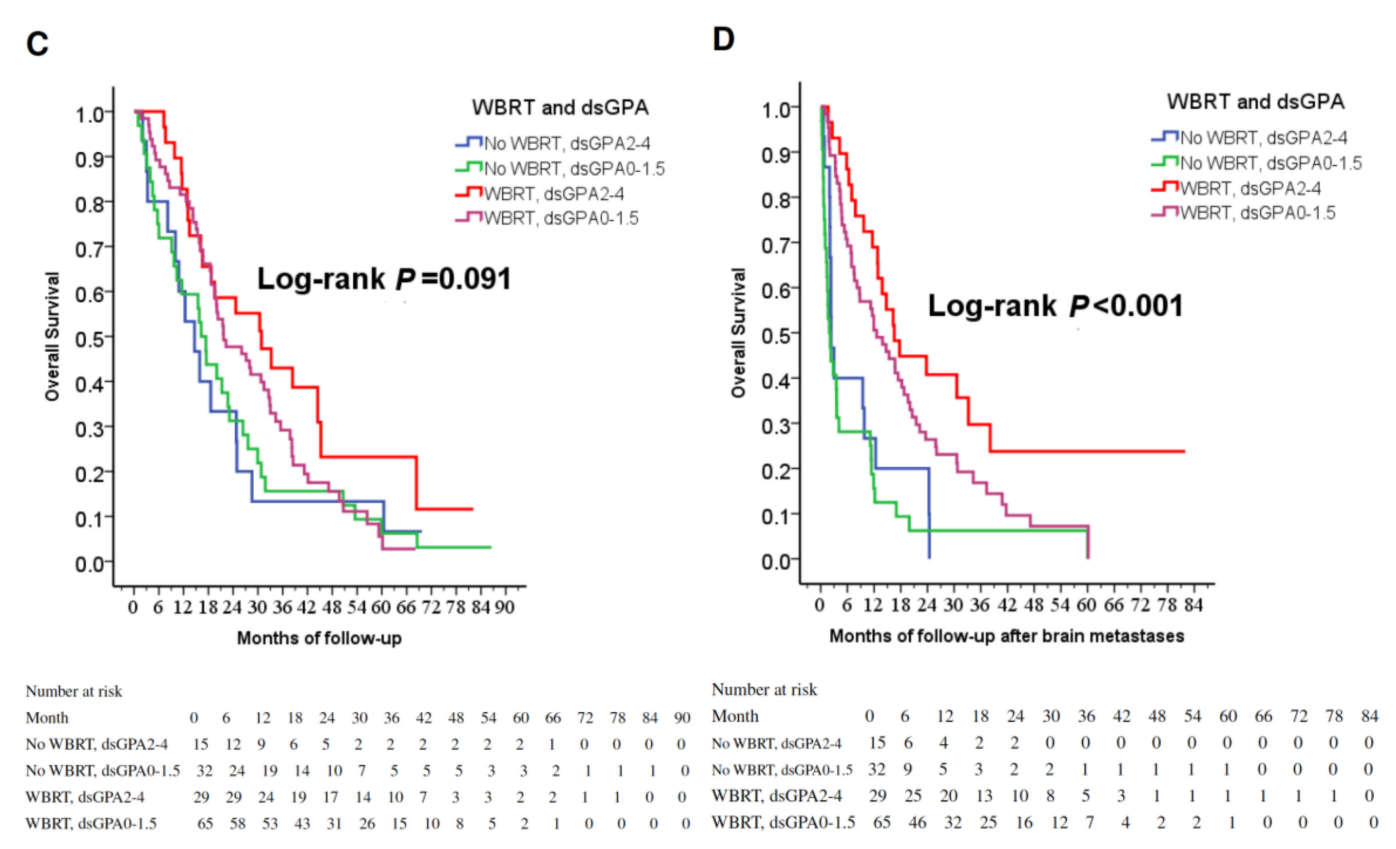
(**A**) Cox regression comparing overall survival in epidermal growth factor receptor-mutant non-small-cell lung cancer patients under tyrosine kinase inhibitors treated with and without WBRT. (**B**) Cox regression comparing overall survival time after the diagnosis of brain metastases in epidermal growth factor receptor-mutant non-small-cell lung cancer patients under tyrosine kinase inhibitors treated with and without WBRT. (**C**) Overall survival of patients stratified by WBRT and dsGPA score. (**D**) Overall survival after the diagnoses of brain metastases in patients stratified by WBRT and dsGPA score. Abbreviations: WBRT=whole-brain radiation therapy; dsGPA=disease-specific Graded Prognostic Assessment.

The 1-year OS rates were 81.9% and 59.6% with and without WBRT (*p* = 0.002). WBRT (*p* = 0.002), younger age (*p* = 0.003), female gender (*p* = 0.029) and surgery to primary lung cancer (*p* = 0.03) were favorable prognostic factors for longer 1-year OS rate (Table 2). WBRT was a favorable prognostic factor for longer OS (*p* = 0.034; Figure 1A). To investigate the prognostic factors, we included five factors with *p* < 0.025 (WBRT, female gender, surgery to primary lung tumor, surgery to BM and smoking status) in a multivariable model (Table 3). WBRT was a strong favorable prognostic factor for longer survival (*p* < 0.001; Figure 1B). 

### 3.3. Subgroup Analyses

In identifying potential differences in the benefits of WBRT for patients by the dsGPA score, there was a trend toward improved OS in the group of TKI + WBRT (*p* = 0.091, Figure 1C); furthermore, WBRT significantly improved OSm regardless of dsGPA score (*p* < 0.001, Figure 1D), while the mean BM-free survival rates were similar in both groups (9.2 ± 13.6 months vs. 14.5 ± 17.8 months, *p* = 0.312). As a result, OSm caused survival difference, and longer OSm contribute to longer OS. WBRT was a strong favorable prognostic factor for longer survival.

## 4. Discussion

We now routinely use molecular selection to identify patients with NSCLC who would benefit from target therapy. The Bureau of National Health Insurance of Taiwan reimburses TKIs prescribed after a diagnosis of stage IIIB or IV lung cancer. Target therapies have resulted in major shifts in the treatment paradigm for lung cancer [30]. Fifteen years ago, Omuro et al. reported that the incidence of the central nervous system as an initial failure site reached 33% in EGFR-TKI responders with advanced NSCLC regardless of disease control in the lungs [31]. Intrinsic resistance of metastatic clones, incomplete TKI penetration of the BBB and longer survival are possible explanations for this high incidence [31]. One retrospective study in Taiwan reported that more patients with advanced EGFR-mutated NSCLC died of BMs than did those with wild-type (44.8% vs. 8.3%, *p* < 0.001) [32]. This change in the causes of death was noted after the era of EGFR-TKI treatment. The present study found that WBRT prolonged OS in patients with EGFR-mutated NSCLC who developed BM. 

Xu et al. stated that aggressive local ablative therapy including surgery or RT to all metastatic sites improved OS compared with local ablative therapy to partial sites or observation alone [33]. Magnuson et al. performed a retrospective study on the topic of the optimal sequence of stereotactic radiosurgery, WBRT, and EGFR-TKIs in patients with EGFR-mutated NSCLC who developed BM. They reported that upfront brain RT resulted in longer OS compared with upfront EGFR-TKIs (stereotactic radiosurgery with 46 months versus WBRT with 30 months versus EGFR-TKI with 25 months, *p* < 0.001) [34]. Li et al. also confirmed the use of upfront WBRT for patients with EGFR-mutated NSCLC and multiple BM improved OS [35]. Although the timing of WBRT was not involved in the present study, we demonstrated worsened OS without WBRT and that WBRT contributed to the addition of approximately one year of survival after the diagnosis of BM.

However, Ke et al. reported no statistically significant difference in the OS between the First-line EGFR-TKI-alone group and First-line EGFR-TKI plus WBRT [36]. It is worth noting that first-generation EGFR-TKIs hardly penetrate across the BBB at the recommended doses [24]. In their study [36], the performance status, dsGPA, surgery to primary or metastatic sites were not documented, and these factors might affect OS. He et al. reported that concurrent EGFR-TKI and WBRT significantly improved the median intracranial progression-free survival compared with EGFR-TKI alone (17.7 vs. 11.0 months, *p* = 0.015); however, there was no significant OS difference (28.1 vs. 24.0 months, *p* = 0.756) [37]. In their study, they prescribed three types of different TKIs (erlotinib, gefitinib and icotinib) and 20 patients in the group of 48 patients who were given EGFR-TKI alone initially received salvage WBRT upon BM progression. This group was not purely without WBRT.

Lee et al. reported that EGFR-mutant NSCLC patients with BM who had received EGFR T790M inhibitors survived longer (41.1 vs. 19.8 months) [38]. Ng et al. found that one of the favorable prognostic factors was female gender (*p* <.001) in patients with NSCLC receiving WBRT [39]. In the present study, the median OSm was 14.3 months (95% CI, 9.5 to 18.3) in the TKI + WBRT group and 2.3 months (95% CI, 2 to 2.6) in the TKI alone group. On multivariate analysis, WBRT (*p* < 0.001) and female (*p* = 0.003) were associated with improved OS.

WBRT is associated with the risks of acute and late toxicities. Cognitive deficits attributed to RT were first reported in children treated for leukemia or brain tumors [40], and this bias was partly caused by greater susceptibility of the developing brain in youngsters [41]. BM by itself negatively affects cognitive function; additionally, baseline cognitive decline from aging in the cancer patients may also impact cognition [42]. Cognitive dysfunction can be caused by brain tumors, psychological distress, comorbidities such as vascular risk factors and diabetes, or by tumor-related epilepsy and its treatment (surgery, RT, anticonvulsants, chemotherapy, or corticosteroids) [40]. It can be difficult to differentiate from the effects of the tumor itself or RT complication [43]. Even though several recent publications have brought into question the role of WBRT and the possible risk of long-term neurotoxicity, WBRT curbed neurological decline [44]. A prospective study showed that the BBB permeability of gefitinib increased in accordance with escalated dose of WBRT [24]. An analysis from Radiation Therapy Oncology Group (RTOG) Study 91-04 showed that WBRT improved the scores on Mini-Mental State Examination in the patients with BM [45]. The optimization of WBRT with pharmacological and technical innovations to selectively spare organs involved in the memory process may decrease the potential long-term neurotoxicity [33]. 

At present, the treatment selection based on driver mutation status improves survival. Given the advancement of systemic therapy for extracranial lesions of metastatic NSCLC, patients now live long enough to develop BM [32]. WBRT, however, may be deferred and even omitted after the emergence of TKI by some clinicians. Based on prospective cohort studies, recently the European Society for Medical Oncology (ESMO) Clinical Practice Guidelines for metastatic NSCLC recommended the use of next-generation TKI for patients with a druggable oncogene driver (EGFR, ALK) and clinically asymptomatic BM [46]. Contradictory results were offered. Another retrospective study in North America reported that First-line WBRT for BM from EGFR/ALK-driven NSCLC was associated with longer time to intracranial progression than was radiosurgery or TKI alone [47]. For patients with ECOG 0-2 in the present study, the absence of WBRT was detrimental to their survival. In terms of different subgroups, even those with favorable dsGPA scores had survival benefit from the addition of WBRT compared with TKI alone.

The results of this study should be interpreted with caution, owing to the heterogeneity of patient characteristics and possible intrinsic bias related to the retrospective design. We intended to minimize bias by using multivariate analyses. There were some pitfalls of the present study. Firstly, radiosurgery was in general not used due to the regulations of National Health Insurance reimbursement. Secondly, we used OS rate to measure the clinical benefits, which might not represent the tumor response. Thirdly, cognitive evaluation was not fully documented. The precise roles of WBRT need to be validated in a randomized control trial. Moreover, Osimertinib is a third-generation EGFR-TKI developed specifically to treat patients with T790M mutation, and only 3.5% of the patients in the study cohort used Osimertinib. 

## 5. Conclusions

The present study suggested that WBRT significantly prolonged OS in patients with EGFR-mutated NSCLC who developed BM. The combination of WBRT and TKI improved OS compared with TKI alone. To the best of our knowledge, this study is the first to demonstrate the negative survival impact from the omission of WBRT in patients with targetable driver mutation. A longer follow-up studying the role of multi-modality treatment in EGFR-mutated NSCLC with BM is urgently warranted.

## Figures and Tables

**Table 1:** Patient characteristics.

	All	WBRT	No WBRT	*p*-Value
No. of cases	141	94	47	
Age				0.112
≦40	5	4	1	
41–70	100	71	29	
>70	36	19	17	
Sex				0.389
Female	88	61	27	
Male	53	33	20	
Initial Clinical stage				0.052 (Fisher)
I–II	8	8	0	
III–IV	133	86	47	
Initial clinical T classification				0.788
1 or 2	38	26	12	
3 or 4	103	68	35	
Initial clinical N classification				0.319
0 or 1	50	36	14	
2 or 3	91	58	33	
Histological grade				0.639
1–2	59	37	22	
3	31	23	8	
NA	51	34	17	
EGFR mutation				
Exon 18	2	1	1	0.557 (Fisher)
Exon 19	63	45	18	0.281
Exon 20	15	8	7	0.247
Exon 21	60	38	22	0.47
NA	5	3	2	0.541 (Fisher)
Lung surgery				0.393
No	121	79	42	
Yes	20	15	5	
Number of lines of systemic chemotherapy				0.078
0–2	104	65	39	
>2	37	29	8	
TKI				
afatinib	17	12	5	0.715
erlotinib	75	52	23	0.474
gefitinib	97	62	35	0.304
osimertinib	5	4	1	0.665 (Fisher)
Number of lines of TKI				0.902
1	89	59	30	
>1	52	35	17	
Mean TKI duration (months ± SD)	17.2 ± 15.6	18.1 ± 15.1	15.4 ± 16.4	0.327
ECOG				0.172
0	71	50	21	
1	62	41	21	
2	8	3	5	
Smoking				0.29
Never	107	75	32	
Former	17	9	8	
Current	17	10	7	
Brain surgery				0.004
No	98	58	40	
Yes	43	36	7	
Symptomatic brain metastases				0.005
No	44	22	22	
Yes	97	72	25	
Size of largest brain tumor				0.046
≦1 cm	50	28	22	
>1 cm	91	66	25	
No. of brain metastases				0.043
1	33	17	16	
2–3	15	13	2	
>3	93	64	29	
Extracranial metastases				
Lung	60	34	26	0.03
Bone	106	69	37	0.491
Liver	24	15	9	0.635
dsGPA				0.898
0.5–1.5	97	65	32	
2–4	44	29	15	

Abbreviations: WBRT: whole-brain radiation therapy; EGFR: Epidermal Growth Factor Receptor; TKI: tyrosine kinase inhibitor; ECOG: Eastern Cooperative Oncology Group; dsGPA: disease-specific Graded Prognostic Assessment. * By *t*-test ** By Chi-square test.

**Table 2:** 1-year overall survival rate.

	No. of Cases	1-Year Survival Rate (Number)	*p*-Value
WBRT			0.002
No	47	59.6% (28)	
Yes	94	81.9% (77)	
Age			0.003
≦40	5	100% (5)	
41–70	100	80% (80)	
>70	36	55.6% (20)	
Gender			0.029
Female	88	80.7% (71)	
Male	53	64.2% (34)	
Initial Clinical stage			0.39
I–II	8	87.5% (7)	
III–IV	138	73.7% (98)	
Clinical Tumor classification			0.386
1 or 2	38	78.9% (30)	
3 or 4	103	72.8% (75)	
Clinical Nodal classification			0.127
0 or 1	50	82% (41)	
2 or 3	91	70.3% (64)	
Extracranial metastases			
Lung	60	78.3% (47)	0.4
Bone	106	71.7% (76)	0.203
Liver	24	70.8% (17)	0.675
Lung surgery			0.03
No	121	71.1% (86)	
Yes	20	95% (19)	
Brain surgery			0.593
No	111	75.7% (84)	
Yes	30	70% (21)	
ECOG			0.299
0	71	76.1% (54)	
1	62	75.8% (47)	
2	8	50% (4)	
Smoking			0.243
Never	107	75.7% (81)	
Former	17	82.4% (14)	
Current	17	58.8% (10)	
Symptomatic brain metastases			0.219
No	44	68.2% (30)	
Yes	97	77.3% (75)	
Size of largest brain tumor			0.357
≦1 cm	50	70% (35)	
>1 cm	91	76.9% (70)	
No. of brain metastases			0.907
1	33	72.7% (24)	
2–3	15	80% (12)	
>3	93	72.7% (69)	
dsGPA			0.821
0.5–1.5	97	75% (72)	
2–4	44	74.2% (33)	

By log-rank test. Abbreviations: WBRT: whole-brain radiation therapy; OS: overall survival; TKI: tyrosine kinase inhibitor; ECOG: Eastern Cooperative Oncology Group; dsGPA: disease-specific Graded Prognostic Assessment.

**Table 3:** Univariate and multivariate Cox regression analyses of covariables associated with OSm.

	Univariate Analyses		Multivariate Analyses	
	HR (95%CI)	*p*-Value	HR (95%CI)	*p*-Value
WBRT				
Yes vs. no	0.36 (0.25 to 0.53)	<0.001	0.34 (0.23 to 0.51)	<0.001
Age				
41–70 vs. ≦40	1.45 (0.46 to 4.58)	0.532		
>70 vs. ≦40	2.69 (0.82 to 8.81)	0.101		
Female vs. male	0.52 (0.36 to 0.75)	0.001	0.44 (0.25 to 0.75)	0.003
Initial Clinical stage				
III–IV vs. I–II	1.77 (0.72 to 4.35)	0.21		
Clinical T classification				
3–4 vs. 1–2	1.26 (0.84 to 1.91)	0.268		
Clinical N classification				
2–3 vs. 0–1	1.31 (0.9 to 1.92)	0.165		
Extracranial metastases				
Lung				
Yes vs. no	1.26 (0.88 to 1.8)	0.216		
Bone				
Yes vs. no	1.38 (0.89 to 2.13)	0.148		
Liver				
Yes vs. no	1.59 (1 to 2.51)	0.046		
Lung surgery				
Yes vs. no	0.5 (0.29 to 0.88)	0.016	0.47 (0.26 to 0.84)	0.01
Brain surgery				
Yes vs. no	0.5 (0.34 to 0.76)	0.001	0.64 (0.41 to 0.97)	0.037
Number of lines of systemic chemotherapy				
>2 vs. 0–2	1.13 (0.76 to 1.69)	0.534		
Number of lines of TKI				
>1 vs. 1	0.71 (0.49 to 1.03)	0.069		
ECOG				
1 vs. 0	0.93 (0.64 to 1.34)	0.693		
2 vs. 0	1.3 (0.59 to 2.84)	0.515		
Smoking				
Former or current vs. never	1.66 (1.11 to 2.48)	0.013	0.85 (0.48 to 1.53)	0.59
Symptomatic brain metastases				
Yes vs. no	0.91 (0.62 to 1.34)	0.639		
Size of largest brain tumor				
>1 cm vs. ≦1 cm	0.99 (0.68 to 1.45)	0.977		
No. of brain metastases				
2–3 vs. 1	1.25 (0.66 to 2.37)	0.5		
>3 vs. 1	1.44 (0.92 to 2.26)	0.109		
dsGPA				
0.5–1.5 vs. 2–4	1.41 (0.95 to 2.1)	0.089		

By Cox regression analyses. Abbreviations: OSm: overall survival time after the diagnosis of brain metastases; WBRT: whole-brain radiation therapy; TKI: tyrosine kinase inhibitor; ECOG: Eastern Cooperative Oncology Group; dsGPA: disease-specific Graded Prognostic Assessment.

## References

[1] Bray F., Ferlay J., Soerjomataram I., Siegel R.L., Torre L.A., Jemal A. (2018). Global cancer statistics 2018: Globocan estimates of incidence and mortality worldwide for 36 cancers in 185 countries. CA Cancer J. Clin..

[2] Walker A.E., Robins M., Weinfeld F.D. (1985). Epidemiology of brain tumors: The national survey of intracranial neoplasms. Neurology.

[3] Hart M.G., Grant R., Walker M., Dickinson H. (2005). Surgical resection and whole brain radiation therapy versus whole brain radiation therapy alone for single brain metastases. Cochrane Database Syst. Rev..

[4] Chao J.H., Phillips R., Nickson J.J. (1954). Roentgen-ray therapy of cerebral metastases. Cancer.

[5] Posner J.B. (1977). Management of central nervous system metastases. Semin. Oncol..

[6] DeAngelis L.M., Delattre J.Y., Posner J.B. (1989). Radiation-induced dementia in patients cured of brain metastases. Neurology.

[7] Patchell R.A., Tibbs P.A., Regine W.F., Dempsey R.J., Mohiuddin M., Kryscio R.J., Markesbery W.R., Foon K.A., Young B. (1998). Postoperative radiotherapy in the treatment of single metastases to the brain: A randomized trial. JAMA.

[8] Chang E.L., Wefel J.S., Hess K.R., Allen P.K., Lang F.F., Kornguth D.G., Arbuckle R.B., Swint J.M., Shiu A.S., Maor M.H. (2009). Neurocognition in patients with brain metastases treated with radiosurgery or radiosurgery plus whole-brain irradiation: A randomised controlled trial. Lancet Oncol..

[9] Kocher M., Soffietti R., Abacioglu U., Villa S., Fauchon F., Baumert B.G., Fariselli L., Tzuk-Shina T., Kortmann R.D., Carrie C. (2011). Adjuvant whole-brain radiotherapy versus observation after radiosurgery or surgical resection of one to three cerebral metastases: Results of the eortc 22952-26001 study. J. Clin. Oncol..

[10] Soffietti R., Kocher M., Abacioglu U.M., Villa S., Fauchon F., Baumert B.G., Fariselli L., Tzuk-Shina T., Kortmann R.D., Carrie C. (2013). A european organisation for research and treatment of cancer phase iii trial of adjuvant whole-brain radiotherapy versus observation in patients with one to three brain metastases from solid tumors after surgical resection or radiosurgery: Quality-of-life results. J. Clin. Oncol..

[11] Churilla T.M., Handorf E., Collette S., Collette L., Dong Y., Aizer A.A., Kocher M., Soffietti R., Alexander B.M., Weiss S.E. (2017). Whole brain radiotherapy after stereotactic radiosurgery or surgical resection among patients with one to three brain metastases and favorable prognoses: A secondary analysis of eortc 22952-26001. Ann. Oncol..

[12] Herbst R.S., Morgensztern D., Boshoff C. (2018). The biology and management of non-small cell lung cancer. Nature.

[13] Li L., Luo S., Lin H., Yang H., Chen H., Liao Z., Lin W., Zheng W., Xie X. (2017). Correlation between egfr mutation status and the incidence of brain metastases in patients with non-small cell lung cancer. J. Thorac. Dis..

[14] Mulvenna P., Nankivell M., Barton R., Faivre-Finn C., Wilson P., McColl E., Moore B., Brisbane I., Ardron D., Holt T. (2016). Dexamethasone and supportive care with or without whole brain radiotherapy in treating patients with non-small cell lung cancer with brain metastases unsuitable for resection or stereotactic radiotherapy (quartz): Results from a phase 3, non-inferiority, randomised trial. Lancet.

[15] Sung S., Lee S.W., Kwak Y.K., Kang J.H., Hong S.H., Kim Y.S. (2018). Intracranial control and survival outcome of tyrosine kinase inhibitor (tki) alone versus tki plus radiotherapy for brain metastasis of epidermal growth factor receptor-mutant non-small cell lung cancer. J. Neuro-Oncol..

[16] Gow C.H., Chien C.R., Chang Y.L., Chiu Y.H., Kuo S.H., Shih J.Y., Chang Y.C., Yu C.J., Yang C.H., Yang P.C. (2008). Radiotherapy in lung adenocarcinoma with brain metastases: Effects of activating epidermal growth factor receptor mutations on clinical response. Clin. Cancer Res..

[17] Eichler A.F., Kahle K.T., Wang D.L., Joshi V.A., Willers H., Engelman J.A., Lynch T.J., Sequist L.V. (2010). Egfr mutation status and survival after diagnosis of brain metastasis in nonsmall cell lung cancer. Neuro Oncol..

[18] Lee H.L., Chung T.S., Ting L.L., Tsai J.T., Chen S.W., Chiou J.F., Leung H.W., Liu H.E. (2012). Egfr mutations are associated with favorable intracranial response and progression-free survival following brain irradiation in non-small cell lung cancer patients with brain metastases. Radiat. Oncol..

[19] Das A.K., Sato M., Story M.D., Peyton M., Graves R., Redpath S., Girard L., Gazdar A.F., Shay J.W., Minna J.D. (2006). Non-small-cell lung cancers with kinase domain mutations in the epidermal growth factor receptor are sensitive to ionizing radiation. Cancer Res..

[20] Qin D.X., Zheng R., Tang J., Li J.X., Hu Y.H. (1990). Influence of radiation on the blood-brain barrier and optimum time of chemotherapy. Int. J. Radiat. Oncol. Biol. Phys..

[21] Van Vulpen M., Kal H.B., Taphoorn M.J., El-Sharouni S.Y. (2002). Changes in blood-brain barrier permeability induced by radiotherapy: Implications for timing of chemotherapy? (review). Oncol. Rep..

[22] Cao Y., Tsien C.I., Shen Z., Tatro D.S., Ten Haken R., Kessler M.L., Chenevert T.L., Lawrence T.S. (2005). Use of magnetic resonance imaging to assess blood-brain/blood-glioma barrier opening during conformal radiotherapy. J. Clin. Oncol..

[23] Khalifa J., Amini A., Popat S., Gaspar L.E., Faivre-Finn C. (2016). International Association for the Study of Lung Cancer Advanced Radiation Technology, C. Brain metastases from nsclc: Radiation therapy in the era of targeted therapies. J. Thorac. Oncol..

[24] Zeng Y.D., Liao H., Qin T., Zhang L., Wei W.D., Liang J.Z., Xu F., Dinglin X.X., Ma S.X., Chen L.K. (2015). Blood-brain barrier permeability of gefitinib in patients with brain metastases from non-small-cell lung cancer before and during whole brain radiation therapy. Oncotarget.

[25] Deng Y., Feng W., Wu J., Chen Z., Tang Y., Zhang H., Liang J., Xian H., Zhang S. (2014). The concentration of erlotinib in the cerebrospinal fluid of patients with brain metastasis from non-small-cell lung cancer. Mol. Clin. Oncol..

[26] Hoffknecht P., Tufman A., Wehler T., Pelzer T., Wiewrodt R., Schutz M., Serke M., Stohlmacher-Williams J., Marten A., Maria Huber R. (2015). Efficacy of the irreversible erbb family blocker afatinib in epidermal growth factor receptor (egfr) tyrosine kinase inhibitor (tki)-pretreated non-small-cell lung cancer patients with brain metastases or leptomeningeal disease. J. Thorac. Oncol..

[27] Yang J.C.-H., Cho B.C., Kim D.-W., Kim S.-W., Lee J.-S., Su W.-C., John T., Kao S.C.-H., Natale R., Goldman J.W. (2017). Osimertinib for patients (pts) with leptomeningeal metastases (lm) from egfr-mutant non-small cell lung cancer (nsclc): Updated results from the bloom study. J. Clin. Oncol..

[28] Sperduto P.W., Chao S.T., Sneed P.K., Luo X., Suh J., Roberge D., Bhatt A., Jensen A.W., Brown P.D., Shih H. (2010). Diagnosis-specific prognostic factors, indexes, and treatment outcomes for patients with newly diagnosed brain metastases: A multi-institutional analysis of 4,259 patients. Int. J. Radiat. Oncol. Biol. Phys..

[29] Edge S.B., Byrd D.R., Compton C.C., Fritz A.G., Frederick L., Greene A.T. (2010). AJCC Cancer Staging Manual.

[30] Mak K.S., Gainor J.F., Niemierko A., Oh K.S., Willers H., Choi N.C., Loeffler J.S., Sequist L.V., Shaw A.T., Shih H.A. (2015). Significance of targeted therapy and genetic alterations in egfr, alk, or kras on survival in patients with non-small cell lung cancer treated with radiotherapy for brain metastases. Neuro Oncol..

[31] Omuro A.M., Kris M.G., Miller V.A., Franceschi E., Shah N., Milton D.T., Abrey L.E. (2005). High incidence of disease recurrence in the brain and leptomeninges in patients with nonsmall cell lung carcinoma after response to gefitinib. Cancer.

[32] Wu W.S., Chen Y.M., Tsai C.M., Shih J.F., Lee Y.C., Perng R.P., Whang-Peng J. (2013). The epidermal growth factor receptor-tyrosine kinase inhibitor era has changed the causes of death of patients with advanced non-small-cell lung cancer. J. Chin. Med. Assoc..

[33] Xu Q., Zhou F., Liu H., Jiang T., Li X., Xu Y., Zhou C. (2018). Consolidative local ablative therapy improves the survival of patients with synchronous oligometastatic nsclc harboring egfr activating mutation treated with first-line egfr-tkis. J. Thorac. Oncol..

[34] Magnuson W.J., Lester-Coll N.H., Wu A.J., Yang T.J., Lockney N.A., Gerber N.K., Beal K., Amini A., Patil T., Kavanagh B.D. (2017). Management of brain metastases in tyrosine kinase inhibitor-naive epidermal growth factor receptor-mutant non-small-cell lung cancer: A retrospective multi-institutional analysis. J. Clin. Oncol..

[35] Li C., Guo J., Zhao L., Hu F., Nie W., Wang H., Zheng X., Shen Y., Gu P., Zhang Y. (2019). Upfront whole brain radiotherapy for multiple brain metastases in patients with egfr-mutant lung adenocarcinoma. Cancer Manag. Res..

[36] Ke S.B., Qiu H., Chen J.M., Shi W., Chen Y.S. (2018). Therapeutic effect of first-line epidermal growth factor receptor tyrosine kinase inhibitor (egfr-tki) combined with whole brain radiotherapy on patients with egfr mutation-positive lung adenocarcinoma and brain metastases. Curr. Med. Sci..

[37] He Z.Y., Li M.F., Lin J.H., Lin D., Lin R.J. (2019). Comparing the efficacy of concurrent egfr-tki and whole-brain radiotherapy vs egfr-tki alone as a first-line therapy for advanced egfr-mutated non-small-cell lung cancer with brain metastases: A retrospective cohort study. Cancer Manag. Res..

[38] Lee J.H., Chen H.Y., Hsu F.M., Chen J.S., Liao W.Y., Shih J.Y., Yu C.J., Chen K.Y., Tsai T.H., Yang J.C. (2019). Cranial irradiation for patients with epidermal growth factor receptor (egfr) mutant lung cancer who have brain metastases in the era of a new generation of egfr inhibitors. Oncologist.

[39] Ng I.W., Tey J.C.S., Chia D.W.T., Yee C.M., Cheo T.S.T. (2019). A review of whole brain radiotherapy outcomes in a high epidermal growth factor receptor mutation rate population: Does quartz apply in Asia?. Asia-Pac. J. Clin. Oncol..

[40] Taphoorn M.J., Klein M. (2004). Cognitive deficits in adult patients with brain tumours. Lancet Neurol..

[41] Crosley C.J., Rorke L.B., Evans A., Nigro M. (1978). Central nervous system lesions in childhood leukemia. Neurology.

[42] Dietrich J., Monje M., Wefel J., Meyers C. (2008). Clinical patterns and biological correlates of cognitive dysfunction associated with cancer therapy. Oncologist.

[43] Schiff D., Wen P.Y., Van den Bent M.J. (2009). Neurological adverse effects caused by cytotoxic and targeted therapies. Nat. Rev. Clin. Oncol..

[44] Loganadane G., Hendriks L., Le Pechoux C., Levy A. (2017). The current role of whole brain radiation therapy in non-small cell lung cancer patients. J. Thorac. Oncol..

[45] Regine W.F., Scott C., Murray K., Curran W. (2001). Neurocognitive outcome in brain metastases patients treated with accelerated-fractionation vs. Accelerated-hyperfractionated radiotherapy: An analysis from radiation therapy oncology group study 91-04. Int. J. Radiat. Oncol. Biol. Phys..

[46] Novello S., Barlesi F., Califano R., Cufer T., Ekman S., Levra M.G., Kerr K., Popat S., Reck M., Senan S. (2016). Metastatic non-small-cell lung cancer: Esmo clinical practice guidelines for diagnosis, treatment and follow-up. Ann. Oncol..

[47] Doherty M.K., Korpanty G.J., Tomasini P., Alizadeh M., Jao K., Labbe C., Mascaux C.M., Martin P., Kamel-Reid S., Tsao M.S. (2017). Treatment options for patients with brain metastases from egfr/alk-driven lung cancer. Radiother. Oncol..

